# Awareness, use, motivations and methods of accessing genetic testing in 2022 in the United States

**DOI:** 10.3389/fgene.2024.1462831

**Published:** 2024-11-08

**Authors:** Sukh Makhnoon, MinJae Lee, Tanushree Prasad, Alexa Badalamenti, Tami Gurley, Erika A. Waters, Celette Sugg Skinner

**Affiliations:** ^1^ Peter O’Donnell Jr. School of Public Health, University of Texas Southwestern Medical Center, Dallas, TX, United States; ^2^ Harold C. Simmons Comprehensive Cancer Center, University of Texas Southwestern Medical Center, Dallas, TX, United States; ^3^ Department of Surgery, Washington University in St. Louis, St Louis, MO, United States

**Keywords:** genetic testing, prevalence, direct-to-consumer, awareness, use, DTC, test use

## Abstract

**Introduction:**

Awareness, access, and use of clinical and direct-to-consumer (DTC) genetic tests has increased in recent years with documented disparities in these services. We provide updated data on test awareness and use, and report novel data on motivations and methods for accessing genetic tests.

**Methods:**

Nationally representative data from the 2022 Health Information National Trends Survey (HINTS 6) were used to assess awareness and use of ancestry, personal trait, specific disease, and carrier testing by sociodemographic characteristics, examine reasons for undergoing tests, and methods of accessing them.

**Results:**

Overall, 81.4% of respondents were aware and 40.0% had undergone testing. Only 10% of tests were ordered by genetic counselors, 80% of carrier and 65% of specific disease tests were ordered by other healthcare providers. Understanding family history was the most common reason for undergoing ancestry (72.2%) or personal trait tests (64.9%) whereas reasons such as doctor’s recommendation (53%–59%), learning more about disease risk (18%–50%), and carrier testing (76%) were common for undergoing disease risk tests and carrier tests. In contrast to ancestry, personal trait, and carrier testing, there were no racial, ethnic, income, or rural/urban difference in use of specific disease risk testing.

**Discussion:**

Diffusion of genetic tests into US society, although incremental, has made sizable increases in awareness, equitable use of specific disease tests but worsening socioeconomic inequality in DTC genetic test use. The study provides update on the state of genetic testing in the US and identifies groups that may need help accessing clinical genomic information and services.

## Introduction

Genetic testing is a predictive and diagnostic tool for various conditions of public health concern including cancer, cardiovascular disease, diabetes, and dementia. Genetic tests can also be used to understand individual ancestry and carrier status for various traits that range from taste of cilantro to serious disease risks. Clinical use of genetic tests has climbed sharply with Medicare payments to laboratories for genetic tests increasing from $351 million in 2016 to $1.9 billion in 2021 before declining to $1.4 billion in 2022 following reports of widespread billing fraud (US Department of Health and Human Services Office of Inspector General, 2023b; US Department of Health and Human Services Office of Inspector General, 2023a). In 2023, the global genetic testing market size was valued at $8.8 billion (Precedence Research, 2024; Grand View Research, 2024; BioSpace, 2024) with North America accounting for 46% (Precedence Research, 2024) of the market with nearly 77,000 genetic tests (BioSpace, 2024). This reflects the technological advancements of genetic testing, availability of new tests, growing adoption of tests in clinical care, and growing demand for direct-to-consumer (DTC) tests. As a result, there is increasing public and research interest in genetic tests offered both in clinical and DTC settings.

Nearly every year since 2000, researchers have estimated the most recent data on awareness and use of genetic testing in the United States. The National Cancer Institute (NCI) and Centers for Disease Control and Prevention routinely collect population-based data on public attitudes towards genetic testing via national surveys such as the National Health Information Survey (Mai et al., 2014) (in 2000, 2005, 2010, and 2011), and the Health Information National Trends Survey (Apathy et al., 2018) (in 2007, 2012, 2013, 2014, and 2020). Periodically, individual research groups also generate similar data from selected provider cohorts (Batra et al., 2002; Kim et al., 2022), clinical samples (Carroll et al., 2020), statewide populations (Goddard et al., 2009), and convenience samples (The Associated Press-NORC Center for Public Affairs Research, 2018). These data help demonstrate the diffusion of innovations such as genetic tests into specific populations or social systems–including, how and which people adopt genetic tests, their test-related health behaviors (Dearing and Cox, 2018). The evidence can inform the field’s purposive dissemination efforts whereby we can take steps to increase clinical genetic tests’ chances of being noticed, accurately perceived, and when necessary, adopted, adapted, and implemented—and, thus, successfully crossing the research-to-practice chasm (Dearing and Kreuter, 2010). Similarly, individuals will be able to make informed decisions on adoption of DTC genetic tests with careful consideration of their perceived utility (Roberts et al., 2017), familial, social, and legal implications (Kilbride and Bradbury, 2020).

Although the expectation that burgeoning genetic knowledge post completion of the human genome project will revolutionize healthcare has long been stymied, public interest in genetics, especially DTC genetics, continues to grow. According to the most recent estimate from 2020, 75% of the U.S. population is aware of genetic testing and 19% have undergone testing themselves (Tiner et al., 2022). The high population awareness is largely attributable to DTC tests, awareness for which has increased from 29% in 2008 (Finney Rutten et al., 2012a) to 75% in 2020 (Tiner et al., 2022) (perhaps in part due to increased advertisement (Schwartz and Woloshin, 2019)). In comparison, awareness of clinical tests such as those for cancer has remained steady, fluctuating between 35% and 44% over the last 20 years (Mai et al., 2014; Tiner et al., 2022). Since awareness is key to the receipt of appropriate genetic tests, these metrics of test awareness and use serve as indicators of the diffusion of genetic discoveries into communities and clinics. Currently, we lack population level data on how different types of genetic tests are accessed by individuals, with a particular paucity of information regarding prenatal genetic carrier tests (Tiner et al., 2022).

In this article, we provide the estimated prevalence of awareness and use of genetic tests in 2022 in the U.S., as well as a comprehensive overview of trends based on up-to-date population-based survey data. We add to the existing literature by examining four types of tests, including information on how different tests are accessed, up-to-date data about disparities in test use by self-reported race, ethnicity, rural/urban status, and income, and discuss their clinical and societal implications.

## Materials and methods

### Data collection

Health Information National Trends Survey (HINTS) is a nationally representative U.S. survey administered by the NCI (Finney Rutten et al., 2020). A detailed overview of the history and methodology used for HINTS data collection is available elsewhere (Finney Rutten et al., 2012b). The most recent administration–HINTS 6 – was fielded March 7 to 8 November 8, 2022 among civilian, non-institutionalized adults aged 18 or older living in the U.S. The survey was administered both on the web and on paper via mail, in English and Spanish, and all groups received $2 pre-paid monetary incentive to participate. Overall, weighted survey response rate was 28.1%. Unless otherwise specified, respondents could check all responses that applied to them.

### Variables and measures

*Awareness and use of genetic testing:* Participants were asked about the types of genetic tests they had (a) *heard of* and (b) *had*. Each question had these six answer choices:

(1) Ancestry testing to understand where you and your relatives come from (for example, tests offered by companies such as Ancestry or 23andMe)
(2) Personal trait testing to understand whether you have genes that are linked to certain characteristic like enjoying the teste of cilantro (for example, tests offered by companies such as Ancestry or 23andMe)
(3) Testing for specific diseases to understand your risk of getting certain diseases such as breast cancer, colon cancer, cardiovascular (heart) disease, diabetes, or dementia/Alzheimer’s
(4) Prenatal genetic carrier testing to determine risk that a man and women will have a baby with certain diseases such as cystic fibrosis or Tay Sachs
(5) Other
(6) None of the above

Tests (1) and (2) were considered DTC tests as clients can directly order them from testing companies, whereas tests (3) and (4) were considered clinical that are administered by a healthcare provider, as defined by the Centers of Disease Control and Prevention (Centers for Disease Control and Prevention, 2024) and HINTS. However, we note that, some DTC tests can provide limited clinical information including three Ashkenazi Jewish founder mutations in *BRCA1*/*2*, carrier status for Beta Thalassemia (e.g., from 23andMe).

The variable on hearing about a genetic test was operationalized as genetic test *awareness* and the variable on having undergone a genetic test was operationalized as genetic test *use*.

*Context of genetic testing:* Respondents who heard about any type of genetic testing were asked “From which of the following sources did you read or hear anything about genetic tests?”: internet (social medical, Google searches), other media (TV, radio, newspaper, magazine), healthcare provider and/or counselor, family or friend. Respondents who underwent any type of genetic test were also asked their reasons for testing, including: doctor’s recommendation, understand my family history, find relatives, learn more about personal traits that may be influenced by genetics, learn more about my risk for certain diseases (for example, cancer or heart disease), understand things like what diet might be best for me, prenatal testing - for example, carrier testing, I received the test as a gift, and other.

Those who specifically “underwent disease risk testing including prenatal carrier tests” were asked how they received the test. Response options were: a genetic counselor ordered the test, my healthcare provider other than a genetic counselor ordered the test, or I ordered the test directly from the laboratory or company on the interest. Because some DTC tests can provide disease risk information, respondents who underwent any type of genetic testing were included in the analysis on method of receiving tests.

*Cancer history:* Respondents were asked whether a first- or second-degree biological relative had ever been diagnosed as having cancer, with response options of yes, no, or not sure.

*Sociodemographic characteristics:* Based on prior research on predictors of genetic testing awareness and use (Mai et al., 2014; Tiner et al., 2022; Agurs-Collins et al., 2015), we included a number of sociodemographic variables in our analysis. These include self-reported age, income, education, self-reported race, ethnicity, sex, employment history, and geography. Some categories were combined because of small sample sizes. Chinese, Filipino, Japanese, Korean, Vietnamese, Asian Indian, and Other Asian were combined into “Asian”; American Indian, Native Hawaiian, Guamanian or Chamorro, Samoan, and Other Pacific Islander were combined into “Indigenous”.

### Statistical analysis

To evaluate associations of various sociodemographic characteristics with genetic test awareness/use, we conducted Chi-square test and multivariable weighted logistic regression analysis. We incorporated survey sampling weights specified for HINTS 6 into our analyses to account for the complex sampling framework used in the HINTS survey and to provide nationally representative estimates of the US population. Response and sociodemographic variables were tabulated and projected over the entire population under the weighted population. Adjusted odds ratios for each sociodemographic variable in relation to each type of genetic test awareness and use were estimated. We used survey procedures in SAS 9.4 (SAS Institute Inc., Cary NC) to perform all statistical analyses and assumed statistical significance at the 0.05 level.

## Results

Of the 6,252 survey respondents, 81.4% (n = 4,674) were aware of genetic testing and 40.0% (n = 1,758) had undergone some type of genetic testing themselves (Table 1).

**TABLE 1:** Awareness and receipt of genetic testing in the US population (unadjusted).

Variable	Category	Awareness of genetic testing n = 5,743 (weighted%)			Receipt of genetic testing n = 4,403 (weighted%)		
		No	Yes n = 4,674	*p*-value	No	Yes n = 1,758	*p*-value
Age (years)	18 to less than 35	125 (14.3)	750 (85.7)	**<0.001**	483 (65.4)	255 (34.6)	**<0.001**
	35 to less than 50	217 (18.5)	953 (81.5)		520 (56)	408 (44)	
	50 to less than 65	314 (18.3)	1,400 (81.7)		830 (61.3)	525 (38.7)	
	65 to less than 75	270 (20.5)	1,048 (79.5)		631 (62.9)	372 (37.1)	
	75 or older	289 (35.6)	523 (64.4)		292 (59.6)	198 (40.4)	
Sex	Female	675 (19.5)	2,792 (80.5)	**0.001**	1,596 (59.1)	1,105 (40.9)	**0.002**
	Male	516 (22.7)	1760 (77.3)		1,114 (65.5)	588 (34.5)	
Education	Less than high school	184 (49.5)	188 (50.5)	**<0.001**	110 (63.6)	63 (36.4)	0.091
	12 years/completed high school	344 (33)	699 (67)		441 (66.8)	219 (33.2)	
	Some college	343 (20.8)	1,303 (79.2)		783 (62.9)	461 (37.1)	
	College graduate or higher	320 (11.9)	2,368 (88.1)		1,379 (59.2)	952 (40.8)	
Race	White	698 (17.9)	3,207 (82.1)	**0.006**	1909 (61.2)	1,208 (38.8)	**0.001**
	Black or African American	264 (26.2)	744 (73.8)		441 (62.6)	264 (37.4)	
	Asian Indian/Asian	102 (33.8)	200 (66.2)		140 (70.7)	58 (29.3)	
	Indigenous	41 (36.3)	72 (63.7)		39 (58.2)	28 (41.8)	
	Multiple races	25 (11.8)	186 (88.2)		95 (51.6)	89 (48.4)	
Ethnicity	Mexican, Mexican American	145 (30.9)	325 (69.1)	**<0.001**	198 (63.3)	115 (36.7)	0.143
	Puerto Rican/Cuban	44 (29.1)	107 (70.9)		61 (61.6)	38 (38.4)	
	Other	118 (36.3)	207 (63.7)		125 (62.8)	74 (37.2)	
	Not Hispanic Ethnicity	795 (17.5)	3,753 (82.5)		2,230 (61.2)	1,413 (38.8)	
	Multiple Hispanic ethnicities	7 (17.9)	32 (82.1)		20 (64.5)	11 (35.5)	
Income	Less than $20,000	363 (38.9)	571 (61.1)	**<0.001**	343 (64.7)	187 (35.3)	**0.006**
	$20,000 to less than $35,000	194 (27.2)	519 (72.8)		332 (67.8)	158 (32.2)	
	$35,000 to less than $50,000	170 (23.6)	551 (76.4)		331 (62.3)	200 (37.7)	
	$50,000 to less than $75,000	147 (15.8)	784 (84.2)		496 (64.8)	270 (35.2)	
	$75,000 or more	231 (10.8)	1909 (89.2)		1,071 (56.9)	810 (43.1)	
Personal history of cancer	No	1,038 (21.2)	3,858 (78.8)	0.118	2,363 (63.3)	1,370 (36.7)	**0.001**
	Yes	166 (18.8)	719 (81.2)		361 (52)	333 (48)	
Family history of cancer	Yes	632 (16.1)	3,300 (83.9)	**<0.001**	1933 (60.4)	1,268 (39.6)	**0.007**
	No	347 (28.2)	882 (71.8)		530 (61.9)	326 (38.1)	
	Not sure	203 (36.1)	359 (63.9)		242 (71.4)	97 (28.6)	
Employment status	Not employed	724 (25.6)	2,100 (74.4)	**<0.001**	1,237 (61.6)	770 (38.4)	0.571
	Employed	470 (16)	2,465 (84)		1,480 (61.4)	929 (38.6)	
Rural-Urban Community Area Code	Metropolitan	1,054 (20.5)	4,092 (79.5)	0.436	2,399 (60.5)	1,564 (39.5)	0.166
	Micropolitan	104 (22.5)	359 (77.5)		215 (63.4)	124 (36.6)	
	Small town	55 (23.4)	180 (76.6)		114 (65.1)	61 (34.9)	
	Rural	33 (26.6)	91 (73.4)		60 (73.2)	22 (26.8)	

^*^ *p*-value for Rao-Scott Chi-square test; significant results are in bold.

Awareness of genetic tests: Overall, 91.9% (n = 5,743) of HINTS respondents answered the question on awareness of genetic tests. Awareness was highest for ancestry testing (71.6%), followed by testing for specific diseases (55.4%), prenatal genetic carrier testing (36.9%), and personal trait testing (25.2%). The most common information source across test types was internet/social media (ranging between 60.5% and 73.1%) and least common was healthcare provider and/or genetic counselor (ranging between 31% and 48.4%).

Multivariable weighted logistic regression analysis for awareness of genetic tests (Table 2) showed six main findings. Awareness was associated with higher odds of being female (OR = 1.58, 95% CI: 1.23–2.02), having a college education (OR = 2.21, 95% CI:1.17–4.16), personal history of cancer (OR = 1.55, 95% CI: 1.16–2.06), and family history of cancer (OR = 1.83, 95% CI: 1.29–2.62). Compared to respondents earning <$20,000 annually, those earning $35k to <$50k, $50k to <$75k and $75k or more were more likely to be aware of genetic testing, which was driven by their high awareness of prenatal carrier tests (Table 2). Awareness was associated with lower odds identifying as non-White, Black, or Asian (OR = 0.49, 95% CI: 0.32–0.77 and OR = 0.18, 95% CI: 0.11–0.30 respectively), and as Hispanic compared to non-Hispanic (OR = 0.48, 95% CI: 0.28–0.80).

**TABLE 2:** Variables associated with genetic test awareness in the US population based on multivariable weighted logistic regression models.

Variable	Category	Ancestry		Personal trait		Specific disease		Prenatal carrier		Total	
		OR	95% CI	OR	95% CI	OR	95% CI	OR	95% CI	OR	95% CI
Age	18 to less than 35	*Ref*		*Ref*		*Ref*		*Ref*		*Ref*	
	35 to less than 50	**0.59**	**0.38–0.91**	1.06	0.76–1.48	**0.62**	**0.43–0.90**	0.78	0.54–1.13	**0.53**	**0.35–0.81**
	50 to less than 65	**0.61**	**0.41–0.90**	**0.64**	**0.42–0.95**	**0.62**	**0.43–0.92**	**0.51**	**0.36–0.73**	**0.57**	**0.36–0.89**
	65 to less than 75	**0.37**	**0.22–0.62**	**0.46**	**0.28–0.77**	**0.39**	**0.25–0.61**	**0.27**	**0.17–0.44**	**0.32**	**0.18–0.56**
	75 or older	**0.14**	**0.08–0.25**	**0.24**	**0.12–0.47**	**0.19**	**0.11–0.34**	**0.15**	**0.09–0.24**	**0.12**	**0.06–0.23**
Sex	Male	*Ref*		*Ref*		*Ref*		*Ref*		*Ref*	
	Female	1.23	0.96–1.58	0.95	0.74–1.21	**1.29**	**1.03–1.63**	**1.55**	**1.21–1.99**	**1.58**	**1.23–2.02**
Education	Less than high school	*Ref*		*Ref*		*Ref*		*Ref*		*Ref*	0.00–0.00
	12 years or completed high school	1.23	0.65–2.32	1.37	0.72–2.58	1.22	0.65–2.28	0.55	0.25–1.18	1.02	0.57–1.83
	Some college	**2.16**	**1.19–3.89**	**2.55**	**1.31–4.95**	1.48	0.79–2.79	0.99	0.47–2.08	1.65	0.93–2.91
	College graduate or higher	**2.76**	**1.41–5.41**	**4.40**	**2.35–8.25**	**2.23**	**1.15–4.30**	**2.08**	**1.01–4.29**	**2.21**	**1.17–4.16**
Race	White	*Ref*		*Ref*		*Ref*		*Ref*		*Ref*	
	Black or African American	**0.35**	**0.25–0.48**	**0.54**	**0.36–0.81**	**0.59**	**0.45–0.78**	**0.57**	**0.41–0.81**	**0.49**	**0.32–0.77**
	Asian	**0.17**	**0.11–0.28**	0.45	0.15–1.34	**0.32**	**0.17–0.61**	**0.39**	**0.17–0.92**	**0.18**	**0.11–0.30**
	Indigenous	**0.56**	**0.27–1.16**	0.75	0.36–1.56	0.78	0.37–1.68	0.79	0.33–1.93	0.55	0.26–1.18
	Multiple races	1.22	0.54–2.73	1.29	0.75–2.21	1.01	0.52–2.00	0.94	0.52–1.68	1.69	0.81–3.54
Ethnicity	Not Hispanic Ethnicity	*Ref*		*Ref*		*Ref*		*Ref*		*Ref*	
	Mexican/M American/Chicano/a	**0.51**	**0.32–0.82**	0.85	0.51–1.39	**0.48**	**0.30–0.76**	0.69	0.40–1.18	**0.48**	**0.28–0.80**
	Puerto Rican/Cuban	**0.55**	**0.32–0.96**	1.19	0.51–2.78	0.88	0.50–1.55	0.93	0.45–1.91	**0.51**	**0.27–0.95**
	Other	**0.28**	**0.18–0.45**	0.89	0.47–1.71	**0.37**	**0.25–0.54**	**0.41**	**0.26–0.64**	**0.35**	**0.22–0.56**
	Multiple Hispanic	0.93	0.22–3.90	2.74	0.47–15.98	1.14	0.25–5.16	1.36	0.23–8.08	0.94	0.16–5.46
Income	Less than 20,000	*Ref*		*Ref*		*Ref*		*Ref*		*Ref*	
	$20,000 to less than $35,000	1.22	0.80–1.86	0.92	0.52–1.64	1.01	0.67–1.52	1.33	0.81–2.18	1.24	0.78–1.97
	$35,000 to less than $50,000	1.63	1.12–2.37	1.10	0.66–1.85	1.17	0.77–1.77	1.29	0.85–1.97	**1.75**	**1.12–2.73**
	$50,000 to less than $75,000	2.26	1.46–3.52	1.52	0.78–2.96	1.42	0.91–2.21	1.45	0.83–2.55	**2.25**	**1.42–3.57**
	$75,000 or more	2.63	1.61–4.31	1.30	0.73–2.32	1.43	0.95–2.16	**1.56**	**1.02–2.38**	**2.88**	**1.82–4.55**
Personal history of cancer	No	*Ref*		*Ref*		*Ref*		*Ref*		*Ref*	
	Yes	1.02	0.78–1.33	1.21	0.87–1.67	**1.59**	**1.25–2.02**	1.17	0.87–1.58	**1.55**	**1.16–2.06**
Family history of cancer	No	*Ref*	0.00–0.00	*Ref*		*Ref*	0.00–0.00	*Ref*		*Ref*	
	Yes	**1.71**	**1.26–2.32**	**1.45**	**1.07–1.97**	**1.44**	**1.08–1.93**	**1.33**	**1.01–1.77**	**1.83**	**1.29–2.62**
	Not sure	0.83	0.51–1.37	1.27	0.78–2.07	0.75	0.46–1.19	0.79	0.50–1.26	0.97	0.55–1.71
Employment status	Not employed	*Ref*		*Ref*		*Ref*		*Ref*		*Ref*	
	Employed	0.82	0.55–1.22	0.87	0.56–1.35	0.99	0.70–1.39	0.83	0.54–1.26	0.91	0.60–1.37
Rural-Urban Community Area Code	Rural	*Ref*		*Ref*		*Ref*		*Ref*		*Ref*	
	Metropolitan	1.44	0.68–3.04	0.76	0.22–2.62	0.80	0.39–1.63	0.66	0.25–1.75	1.23	0.65–2.34
	Micropolitan	0.95	0.40–2.28	0.63	0.19–2.17	0.93	0.40–2.14	0.85	0.31–2.36	0.99	0.41–2.37
	Small town	1.58	0.63–3.97	0.83	0.24–2.88	0.65	0.24–1.78	0.41	0.13–1.27	1.06	0.41–2.77

OR: weighted odds ratio; CI: weighted confidence interval; significant results are in bold.

**FIGURE 1:**
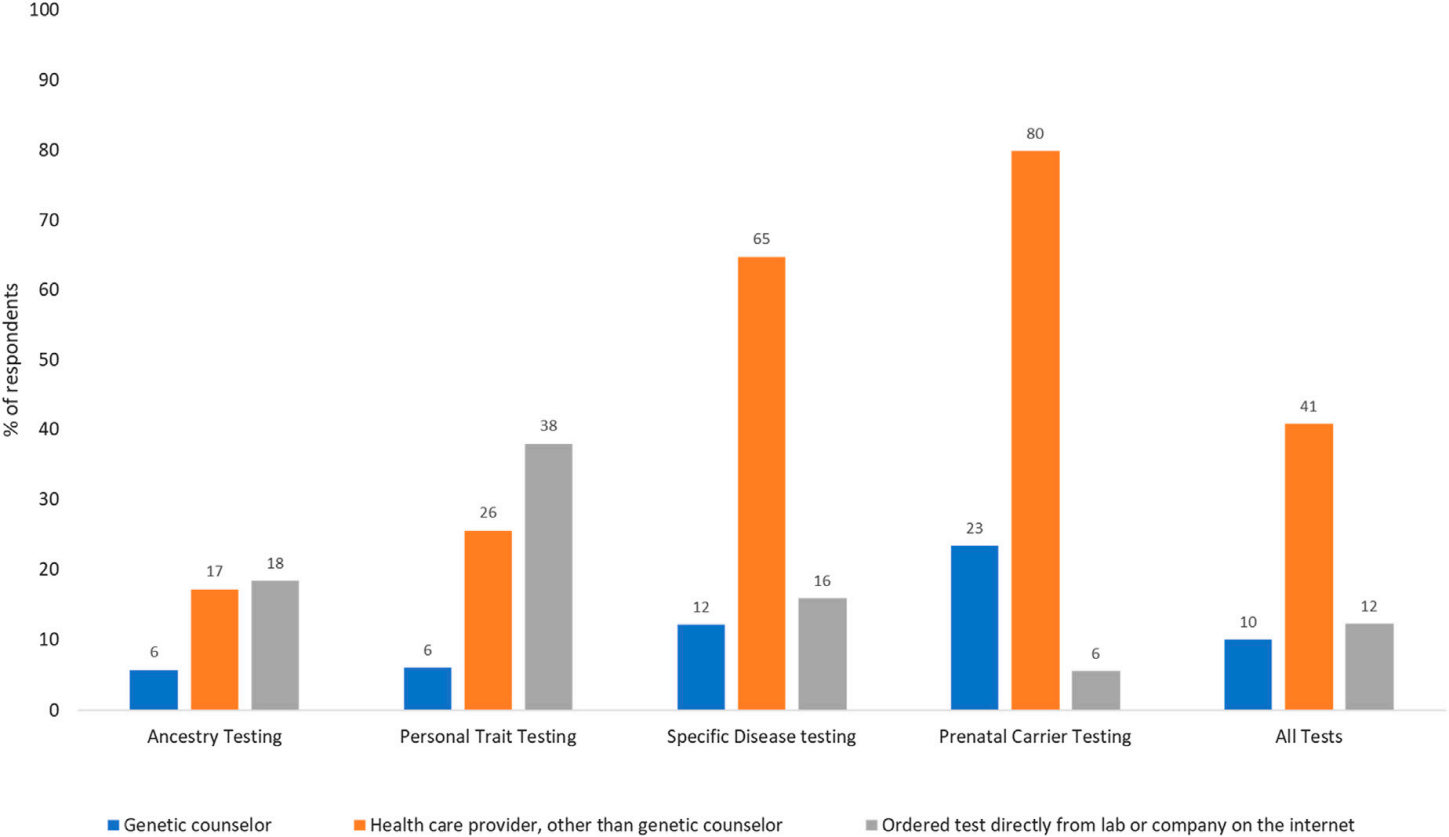
Methods of accessing genetic testing for disease risk in the US population (N = 1,009; weighted %s). Percentages may not sum up to 100 as selecting multiple answer choices was allowed in this question. The fourth answer choice was not having undergone any genetic test.

**FIGURE 2:**
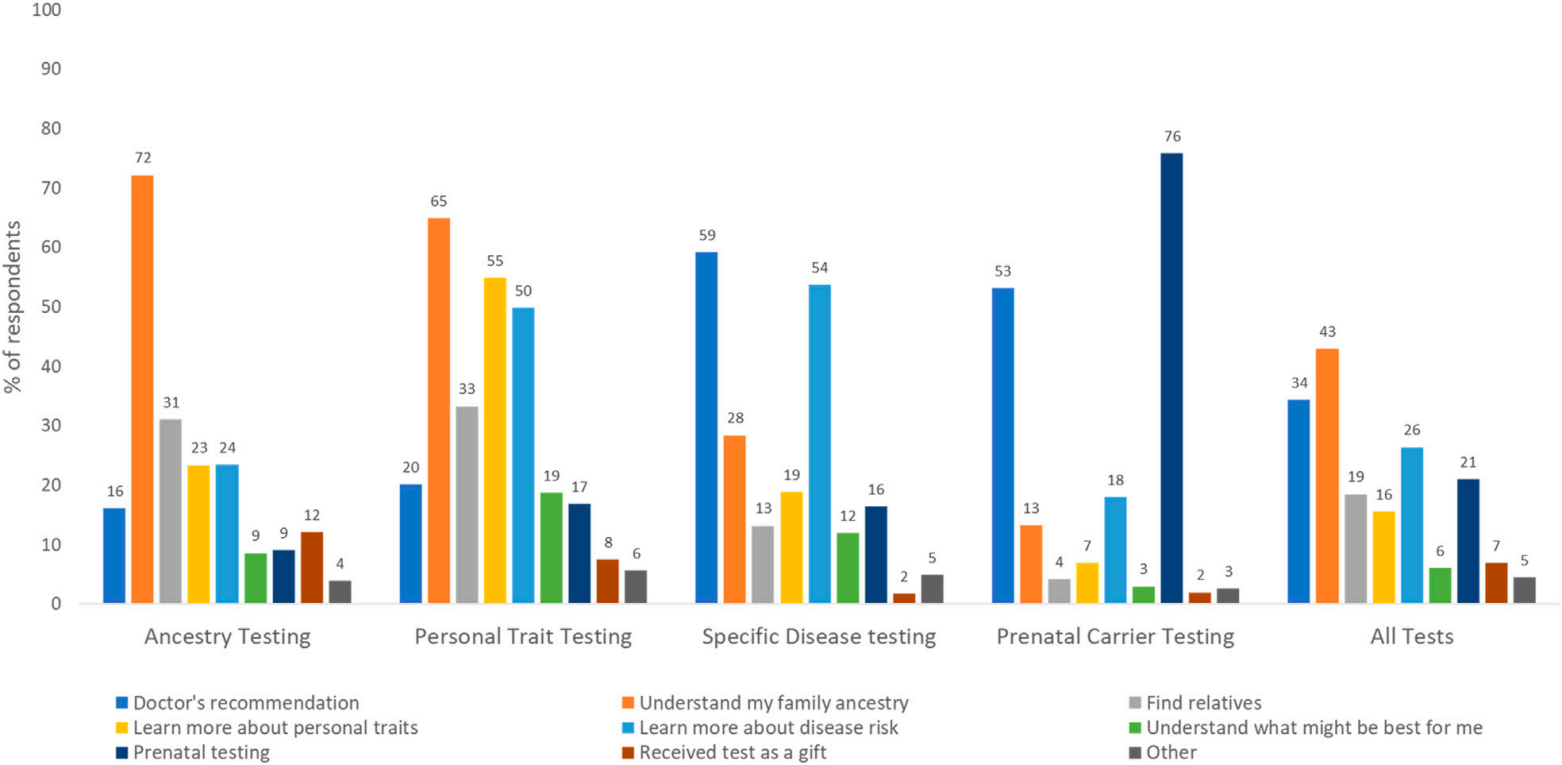
Rationale for undergoing different types of genetic tests as reported by survey respondents (N = 1,758; weighted %s). Percentages may not sum up to 100 as selecting multiple answer choices was allowed in this question.

Use of genetic tests: Of the 4,403 respondents who answered the question on test use, 40% had undergone some type of genetic testing. Use was highest for ancestry (22.6%), followed by specific disease risk, carrier, and personal trait testing (15.9%, 7.8% and 6.2% respectively). Of all respondents, 5.4% had undergone two tests, 2% had undergone three tests, 0.5% had undergone four tests, and <0.01% had undergone all five genetic tests. As shown in Figure 1, only 10% of all tests were ordered by genetic counselors. The majority of prenatal carrier (80%) and specific disease tests (65%) were ordered by a healthcare provider other than a genetic counselor, and personal trait tests were commonly ordered directly from the laboratory or company on the internet (38%). Participants were asked about their rationale for undergoing genetic tests and had the option to select more than one response (Figure 2). Understanding family history was the most common reason for undergoing ancestry or personal trait tests (72.2% and 64.9% respectively) whereas reasons such as doctor’s recommendation (53%–59%), learning more about disease risk (18%–50%), and prenatal carrier testing (76%) were common for undergoing disease risk and prenatal carrier tests respectively (Figure 2).

Table 3 shows the bivariate and Table 4 shows multivariable associations of test use. Notably, females were more likely to undergo testing than males (OR = 1.33, 95% CI: 1.08–1.63) driven by higher use of prenatal carrier testing. Individuals with personal history of cancer were more likely to undergo testing than those without (OR = 1.51, 95% CI: 1.07–2.13), which was largely attributable to higher use of specific disease tests (OR = 2.49, 95% CI: 1.69–3.67). Respondents who self-identified as multiracial were more likely to undergo testing than White respondents, the largest group of respondents, (OR = 2.37, 95% CI: 1.39–4.06), a trend driven by higher use of ancestry tests and personal trait tests in this group. Compared to White respondents, Black respondents were less likely to undergo personal trait testing (OR = 0.55, 95% CI: 0.31–0.96) and Asian respondents were less likely to undergo ancestry testing (OR = 0.42, 95% CI: 0.23–0.78).

**TABLE 3:** Sociodemographic distribution of genetic test use (unadjusted).

Variable	Category	Ancestry		Personal trait		Specific disease		Prenatal carrier		Total	
		n (weighted %)		n (weighted %)		n (weighted %)		n (weighted %)		n (weighted %)	
		No	Yes	No	Yes	No	Yes	No	Yes	No	Yes
Age (years)	18 to less than 35	596 (80.8)	142 (19.2)	679 (92)	59 (8)	658 (89.2)	80 (10.8)	649 (87.9)	89 (12.1)	483 (65.4)	255 (34.6)
	35 to less than 50	729 (78.6)	199 (21.4)	851 (91.7)	77 (8.3)	771 (83.1)	157 (16.9)	760 (81.9)	168 (18.1)	520 (56)	408 (44)
	50 to less than 65	1,060 (78.2)	295 (21.8)	1,273 (93.9)	82 (6.1)	1,109 (81.8)	246 (18.2)	1275 (94.1)	80 (5.9)	830 (61.3)	525 (38.7)
	65 to less than 75	760 (75.8)	243 (24.2)	958 (95.5)	45 (4.5)	830 (82.8)	173 (17.2)	981 (97.8)	22 (2.2)	631 (62.9)	372 (37.1)
	75 or older	347 (70.8)	143 (29.2)	472 (96.3)	18 (3.7)	416 (84.9)	74 (15.1)	479 (97.8)	11 (2.2)	292 (59.6)	198 (40.4)
Sex	Female	2092 (77.5)	609 (22.5)	2,531 (93.7)	170 (6.3)	2,222 (82.3)	479 (17.7)	2,424 (89.7)	277 (10.3)	1,596 (59.1)	1,105 (40.9)
	Male	1,316 (77.3)	386 (22.7)	1,600 (94)	102 (6)	1,479 (86.9)	223 (13.1)	1,632 (95.9)	70 (4.1)	1,114 (65.5)	588 (34.5)
Education	Less than high school	150 (86.7)	23 (13.3)	165 (95.4)	8 (4.6)	138 (79.8)	35 (20.2)	160 (92.5)	13 (7.5)	110 (63.6)	63 (36.4)
	12 years/completed high school	556 (84.2)	104 (15.8)	640 (97)	20 (3)	544 (82.4)	116 (17.6)	627 (95)	33 (5)	441 (66.8)	219 (33.2)
	Some college	967 (77.7)	277 (22.3)	1,180 (94.9)	64 (5.1)	1,040 (83.6)	204 (16.4)	1,170 (94.1)	74 (5.9)	783 (62.9)	461 (37.1)
	College graduate or higher	1738 (74.6)	593 (25.4)	2,153 (92.4)	178 (7.6)	1982 (85)	349 (15)	2,105 (90.3)	226 (9.7)	1,379 (59.2)	952 (40.8)
Race	White	2,351 (75.4)	766 (24.6)	2,914 (93.5)	203 (6.5)	2,657 (85.2)	460 (14.8)	2,873 (92.2)	244 (7.8)	1909 (61.2)	1,208 (38.8)
	Black or African American	598 (84.8)	107 (15.2)	677 (96)	28 (4)	553 (78.4)	152 (21.6)	658 (93.3)	47 (6.7)	441 (62.6)	264 (37.4)
	Asian Indian/Asian	172 (86.9)	26 (13.1)	187 (94.4)	11 (5.6)	179 (90.4)	19 (9.6)	179 (90.4)	19 (9.6)	140 (70.7)	58 (29.3)
	Other	51 (76.1)	16 (23.9)	62 (92.5)	5 (7.5)	54 (80.6)	13 (19.4)	64 (95.5)	3 (4.5)	39 (58.2)	28 (41.8)
	Multiple races selected	121 (65.8)	63 (34.2)	166 (90.2)	18 (9.8)	149 (81)	35 (19)	165 (89.7)	19 (10.3)	95 (51.6)	89 (48.4)
Ethnicity	Mexican	250 (79.9)	63 (20.1)	286 (91.4)	27 (8.6)	266 (85)	47 (15)	279 (89.1)	34 (10.9)	198 (63.3)	115 (36.7)
	Puerto Rican/Cuban	83 (83.8)	16 (16.2)	94 (94.9)	5 (5.1)	80 (80.8)	19 (19.2)	86 (86.9)	13 (13.1)	61 (61.6)	38 (38.4)
	Indigenous	159 (79.9)	40 (20.1)	184 (92.5)	15 (7.5)	166 (83.4)	33 (16.6)	184 (92.5)	15 (7.5)	125 (62.8)	74 (37.2)
	Not Hispanic Ethnicity	2,794 (76.7)	849 (23.3)	3,424 (94)	219 (6)	3,068 (84.2)	575 (15.8)	3,364 (92.3)	279 (7.7)	2,230 (61.2)	1,413 (38.8)
	Multiple Hispanic ethnicities	23 (74.2)	8 (25.8)	29 (93.5)	2 (6.5)	27 (87.1)	4 (12.9)	29 (93.5)	2 (6.5)	20 (64.5)	11 (35.5)
Income	Less than $20,000	446 (84.2)	84 (15.8)	503 (94.9)	27 (5.1)	422 (79.6)	108 (20.4)	498 (94)	32 (6)	343 (64.7)	187 (35.3)
	$20,000 to less than $35,000	410 (83.7)	80 (16.3)	473 (96.5)	17 (3.5)	422 (86.1)	68 (13.9)	466 (95.1)	24 (4.9)	332 (67.8)	158 (32.2)
	$35,000 to less than $50,000	420 (79.1)	111 (20.9)	503 (94.7)	28 (5.3)	456 (85.9)	75 (14.1)	490 (92.3)	41 (7.7)	331 (62.3)	200 (37.7)
	$50,000 to less than $75,000	607 (79.2)	159 (20.8)	727 (94.9)	39 (5.1)	653 (85.2)	113 (14.8)	716 (93.5)	50 (6.5)	496 (64.8)	270 (35.2)
	$75,000 or more	1,358 (72.2)	523 (27.8)	1725 (91.7)	156 (8.3)	1,575 (83.7)	306 (16.3)	1,685 (89.6)	196 (10.4)	1,071 (56.9)	810 (43.1)
Personal history of cancer	No	2,915 (78.1)	818 (21.9)	3,500 (93.8)	233 (6.2)	3,211 (86)	522 (14)	3,415 (91.5)	318 (8.5)	2,363 (63.3)	1,370 (36.7)
	Yes	510 (73.5)	184 (26.5)	655 (94.4)	39 (5.6)	510 (73.5)	184 (26.5)	664 (95.7)	30 (4.3)	361 (52)	333 (48)
Family history of cancer	Yes	2,444 (76.4)	757 (23.6)	3,012 (94.1)	189 (5.9)	2,677 (83.6)	524 (16.4)	2,947 (92.1)	254 (7.9)	1933 (60.4)	1,268 (39.6)
	No	679 (79.3)	177 (20.7)	795 (92.9)	61 (7.1)	720 (84.1)	136 (15.9)	779 (91)	77 (9)	530 (61.9)	326 (38.1)
	Not sure	280 (82.6)	59 (17.4)	317 (93.5)	22 (6.5)	297 (87.6)	42 (12.4)	323 (95.3)	16 (4.7)	242 (71.4)	97 (28.6)
Employment status	Not employed	1,532 (76.3)	475 (23.7)	2,229 (92.5)	180 (7.5)	1,674 (83.4)	333 (16.6)	1910 (95.2)	97 (4.8)	1,237 (61.6)	770 (38.4)
	Employed	1884 (78.2)	525 (21.8)	1915 (95.4)	92 (4.6)	2038 (84.6)	371 (15.4)	2,160 (89.7)	249 (10.3)	1,480 (61.4)	929 (38.6)
Rural-Urban Community Area Code	Metropolitan	3,043 (76.8)	920 (23.2)	3,714 (93.7)	249 (6.3)	3,324 (83.9)	639 (16.1)	3,632 (91.6)	331 (8.4)	2,399 (60.5)	1,564 (39.5)
	Micropolitan	277 (81.7)	62 (18.3)	319 (94.1)	20 (5.9)	282 (83.2)	57 (16.8)	316 (93.2)	23 (6.8)	215 (63.4)	124 (36.6)
	Small town	141 (80.6)	34 (19.4)	166 (94.9)	9 (5.1)	141 (80.6)	34 (19.4)	162 (92.6)	13 (7.4)	114 (65.1)	61 (34.9)
	Rural	69 (84.1)	13 (15.9)	79 (96.3)	3 (3.7)	75 (100)	(0)	78 (95.1)	4 (4.9)	60 (73.2)	22 (26.8)

**TABLE 4:** Variables associated with genetic testing use based on multivariable weighted logistic regression models.

Variable	Category	Ancestry		Personal trait		Specific disease		Prenatal carrier		Total	
		OR	95% CI	OR	95% CI	OR	95% CI	OR	95% CI	OR	95% CI
Age (years)	18 to less than 35	*Ref*		*Ref*		*Ref*		*Ref*		*Ref*	
	35 to less than 50	1.44	0.91–2.27	1.07	0.62–1.85	1.54	0.86–2.75	1.76	0.93–3.34	**1.66**	**1.13–2.44**
	50 to less than 65	**1.62**	**1.08–2.43**	0.97	0.50–1.87	1.44	0.76–2.75	**0.53**	**0.28–0.98**	1.28	0.91–1.81
	65 to less than 75	**1.84**	**1.17–2.90**	**0.44**	**0.20–0.95**	1.05	0.52–2.10	**0.14**	**0.06–0.31**	1.19	0.77–1.84
	75 or older	**2.73**	**1.59–4.71**	0.56	0.18–1.78	1.02	0.43–2.41	0.45	0.06–3.55	**1.82**	**1.09–3.03**
Sex	Male	*Ref*		*Ref*		*Ref*		*Ref*		*Ref*	
	Female	0.99	0.79–1.24	1.19	0.84–1.70	1.39	0.99–1.95	**2.90**	**2.04–4.13**	**1.33**	**1.08–1.63**
Education	Less than high school	*Ref*		*Ref*		*Ref*		*Ref*		*Ref*	
	12 years or completed high school	1.21	0.53–2.76	0.44	0.14–1.35	1.61	0.80–3.24	0.26	0.02–3.52	0.91	0.29–2.90
	Some college	1.74	0.96–3.17	1.31	0.53–3.22	1.31	0.64–2.71	0.45	0.04–4.98	1.11	0.38–3.22
	College graduate or higher	2.05	1.03–4.07	1.89	0.73–4.91	1.17	0.62–2.22	0.78	0.06–9.77	1.24	0.41–3.75
Race	White	*Ref*		*Ref*		*Ref*		*Ref*		*Ref*	
	Black or African American	0.78	0.51–1.19	**0.55**	**0.31–0.96**	1.44	0.92–2.25	1.14	0.68–1.91	1.20	0.88–1.63
	Asian	**0.42**	**0.23–0.78**	0.48	0.18–1.26	0.46	0.21–1.02	0.69	0.35–1.37	**0.60**	**0.40–0.90**
	Indigenous	1.09	0.42–2.83	1.92	0.22–16.71	0.46	0.21–1.02	0.15	0.02–1.56	1.43	0.61–3.35
	Multiple races	**2.23**	**1.11–4.46**	**2.83**	**1.60–5.01**	1.92	0.96–3.85	1.48	0.49–4.52	**2.37**	**1.39–4.06**
Ethnicity	Not Hispanic	*Ref*		*Ref*		*Ref*		*Ref*		*Ref*	
	Mexican/M. American/Chicano/a	1.13	0.55–2.30	1.70	0.56–5.14	0.66	0.32–1.36	1.01	0.51–2.00	1.01	0.59–1.72
	Puerto Rican/Cuban	0.49	0.16–1.52	2.66	0.11–61.62	2.83	0.87–9.24	2.61	0.53–12.86	**2.27**	**1.03–5.03**
	Other	1.05	0.57–1.93	1.46	0.53–4.02	1.03	0.49–2.15	0.57	0.16–2.10	0.79	0.44–1.43
	Multiple Hispanic ethnicities	1.83	0.59–5.71	0.31	0.02–5.01	0.19	0.02–1.84	0.44	0.09–2.09	0.90	0.26–3.11
Income	Less than $20,000	*Ref*		*Ref*		*Ref*		*Ref*		*Ref*	
	$20,000 to less than $35,000	0.94	0.47–1.87	0.71	0.26–1.90	0.55	0.29–1.04	2.12	0.39–11.55	0.94	0.51–1.74
	$35,000 to less than $50,000	1.64	0.82–3.25	1.57	0.56–4.38	1.03	0.47–2.25	1.46	0.52–4.11	1.40	0.80–2.45
	$50,000 to less than $75,000	1.13	0.57–2.22	1.07	0.40–2.85	0.64	0.37–1.13	0.78	0.25–2.49	0.74	0.45–1.20
	$75,000 or more	**2.05**	**1.16–3.62**	1.30	0.54–3.13	0.77	0.47–1.27	1.20	0.40–3.64	1.25	0.82–1.91
Personal history of cancer	No	*Ref*		*Ref*		*Ref*		*Ref*		*Ref*	
	Yes	1.05	0.78–1.42	1.35	0.59–3.10	**2.49**	**1.69–3.67**	0.62	0.25–1.57	**1.51**	**1.07–2.13**
Family history of cancer	No	*Ref*		*Ref*		*Ref*		*Ref*		*Ref*	
	Yes	1.03	0.74–1.43	**0.51**	**0.31–0.82**	1.17	0.76–1.82	0.86	0.56–1.32	1.01	0.73–1.38
	Not sure	0.63	0.35–1.14	0.61	0.27–1.41	0.73	0.35–1.50	0.84	0.19–3.67	0.63	0.34–1.19
Employment status	Not employed	*Ref*		*Ref*		*Ref*		*Ref*		*Ref*	
	Employed	1.07	0.75–1.51	1.38	0.77–2.47	1.13	0.73–1.75	1.30	0.67–2.52	1.19	0.85–1.67
Rural-Urban Community Area Code	Rural	*Ref*		*Ref*		*Ref*		*Ref*		*Ref*	
	Metropolitan	1.43	0.51–4.00	1.25	0.06–24.91	1.11	0.39–3.17	1.22	0.65–2.29	1.25	0.59–2.67
	Micropolitan	1.27	0.37–4.37	1.51	0.07–32.90	1.61	0.51–5.04	2.72	0.80–9.21	2.23	0.88–5.64
	Small town	1.78	0.59–5.34	2.47	0.10–62.97	1.43	0.38–5.31	0.87	0.28–2.71	1.33	0.62–2.84

OR: weighted odds ratio; CI: weighted confidence interval.

## Discussion

From 2020 (publication of the last HINTS report) to 2022 (Tiner et al., 2022), awareness of genetic testing has increased (75%–81%) and use of genetic test has doubled (19%–40%) (Tiner et al., 2022). High test awareness in 2022 was largely attributable to high awareness of ancestry testing (71.6%) as well as high awareness of disease specific testing (55%). It has been previously suggested that awareness of ancestry testing may be (Rollins et al., 2014) attributable to increased advertisement of these tests to consumers (Schwartz and Woloshin, 2019; Carlson, 2009). A new finding is the high awareness of disease specific tests, which include tests for cancer (that comprise the largest market share of all clinical genetic tests), cardiovascular diseases, and neurodevelopmental disorders. The increased awareness of cancer genetic tests among those with personal and family history of cancer is encouraging as these factors confer increased familial cancer risk and in some cases individuals with these factors may benefit from genetic cancer risk evaluation. For DTC genetic testing, survey results suggested higher awareness among more educated and higher income groups and lower awareness among racial and ethnic minorities, consistent with prior evidence that awareness of DTC genetic tests has yet to reach individuals of all socioeconomic groups (Tiner et al., 2022). As more evidence emerges about whether DTC testing is beneficial, it will be important to disseminate this information among all population groups. Differences in awareness must be addressed through purposeful dissemination strategies to narrow the gap in implementation of those genomic medicine services that are backed by evidence for clinical utility. However, the previously reported rural/urban difference in test awareness (Salloum et al., 2018) was not evident in the data, perhaps due to the fact that the most common information source across test types was internet/social media–a communication channel relatively easily accessed by individuals from a variety of geographic locations.

Test use largely mirrored the pattern of test awareness. Unlike previous reports, where individuals with higher income were more likely to undergo genetic testing, we only observed this pattern of association for Ancestry testing among individuals earning $75,000 or more per year. In contrast to previous studies (Carroll et al., 2020), the disparity was primarily observed in the use of DTC tests such as ancestry tests and personal trait tests. Compared to White respondents, Asian and Black respondents were less likely to undergo DTC ancestry and personal trait testing. The higher use of these DTC tests among multi-racial individuals may either indicate that individuals underwent testing to discover the genetic basis of their genealogical mixed race or that undergoing a genetic ancestry test caused them to respond to questions on race/ethnicity differently (Johfre et al., 2021). The lack of income difference in specific disease risk testing may be attributed, in part, to subsidized testing offered at many laboratories through financial assistance programs and sponsored testing (Invitae, 2024), inclusive insurance coverage policies (private payors and Medicare base coverage policies on national guidelines), and low out-of-pocket costs. Although some racial and ethnic disparities in overall genetic test use remain, there was no racial or ethnic difference in use of specific disease risk tests.

Only a small proportion of all genetic tests (10%) involved genetic counselors as the ordering provider. Even for genetic tests for specific diseases that require healthcare provider involvement, most tests (64.8%) were ordered by providers other than genetic counselors. This likely documents the increasing use of point of care testing by clinicians, or mainstreaming, which is necessary for wider genomic care delivery. Still, without the involvement of genetic counselors, who are trained to provide support and deliver genomic healthcare holistically, it is crucial that providers receive continued genetic education to help patients understand, adapt, and adjust to the medical or psychosocial consequences of genetic information, and manage patient care based on evolving guidelines. In contrast to DTC tests, that are available to any self-paying customer, clinical genetic tests are only available when specific guideline-recommended testing criteria are met and are usually paid through insurance. Understanding family history was a commonly reported reason for undergoing DTC tests. This combined with higher DTC test use among multiracial respondents points to the growing public interest in genetic tests as a method of understanding genealogical history.

In examining patterns of test awareness and use, it is important to distinguish DTC health-risk tests from high-risk disease tests, as their motivations and implications are vastly different. Some DTC tests (e.g., 23andMe) may report SNP-level associations to several disease-associated loci, whereas clinical tests are designed to test for disease-specific causal variants. However, because DTC tests can sometimes include results from specific causal variants, e.g., three Ashkenazi Jewish founder mutations in *BRCA1*/*2*, it is unclear whether respondents who selected “testing for specific diseases” underwent DTC tests or clinical tests. This makes it challenging to compare the 2020 and 2022 HINTS survey findings. Still, if we categorize tests where 23andMe is specified as an example as a DTC test, we find that DTC health risk test awareness has halved (52%–25.2%) but use has remained stable (6%–6.2%) since 2020. However, awareness of ancestry testing, that does not provide health-risk information, has remained stable: 71% in 2020 to 71.6% in 2022; and use has increased from 14% to 22.6%. By disentangling DTC health risk tests from AncestryDNA, the highest advertiser of all DTC laboratory tests (spent $38 million in 2016 to promote genealogy and ethnicity DNA tests (Schwartz and Woloshin, 2019)), we are beginning to understand the population reach of these two types of tests. In contrast, clinical genetic testing for specific diseases (which in 2020 only included “High risk cancer testing for example, *BRCA1/2* or Lynch Syndrome”) has increased from 36% to 55% and use has increased from 5% to 16%.

Diffusion of innovations (Dearing and Cox, 2018) such as genetic tests, changes societies over time. Currently, these changes manifest as differences in awareness of genetic tests and increasing socioeconomic inequality in DTC test use in the US. For clinical testing, the diffusion has been more equitable, aided by factors such as prominent early adopters of genetic tests (Borzekowski et al., 2014), decreasing cost of genetic tests, specific dissemination efforts informed by guidelines (Daly et al., 2021), and with some exceptions (Whitworth et al., 2017), more inclusive insurance reimbursement policies. However, inequities in testing among minoritized populations, although not observed here, warrant careful monitoring using data sources that are not subject to response bias. The diffusion of clinically important genetic tests within eligible populations has the potential to increase access and reduce disparities in clinical genetic testing. Conversely, widespread use of DTC genetic tests raises a series of concerns including privacy issues, providing “reassuring” false negatives, and burdening the healthcare system given the need for clinical confirmation of these test results that require healthcare provider involvement.

Strengths of this study include data from a population-based survey, use of survey weights to obtain population representative numbers, and the ability to compare results from this latest survey to past data. Limitations include the relatively low response rate (28.1%) which may limit the extent to which the results are representative of the US population. As with any self-reported data, may be subject to social desirability bias. In particular, the question on who ordered a test may have been misinterpreted as genetic counselors would not be expected to be involved in ordering DTC tests. Even for clinical tests, genetic counselors may more typically facilitate testing on behalf of the ordering provider rather than ordering the test themselves, a difference that is likely imperceptible to most patients/consumers. Due to the small sample sizes in some categories (<25), some results may yield unreliable estimates and should be interpreted with care. The cross-sectional nature of survey precluded the ability to draw conclusion about causality between outcomes of interest and sociodemographic and clinical characteristics. However, our study provides important up-to-date data on the current state of genetic testing in the US, an important step towards identifying groups that may need help accessing and using genetic tests.

In conclusion, in this 2022 US population-based survey, we found higher prevalence in awareness and use of genetic tests than previously reported and different patterns of associations with socio-demographic characteristics than the previous (2020) survey. We extend prior work from nationally representative US data on genetic testing, through reporting on prenatal carrier testing as well as examining reasons for undergoing tests, and methods of accessing them. Diffusion of genetic tests, although incremental, has made sizable increases in awareness and use between 2000 and 2022. The study provides update on the state of genetic testing in the US and identifies groups that may need help accessing clinical genomic information and services.

## Data Availability

Publicly available datasets were analyzed in this study. This data can be found here: HINTS Dataset https://hints.cancer.gov/data/default.aspx.
